# Dataset of thermographic images for the detection of buried landmines

**DOI:** 10.1016/j.dib.2023.109443

**Published:** 2023-07-24

**Authors:** Hermes Alejandro Tenorio-Tamayo, Juan Camilo Forero-Ramírez, Bryan García, Humberto Loaiza-Correa, Andrés David Restrepo-Girón, Sandra Esperanza Nope-Rodríguez, Asfur Barandica-López, José Tomás Buitrago-Molina

**Affiliations:** Escuela de Ingeniería Eléctrica y Electrónica (EIEE), Facultad de Ingeniería, Universidad del Valle, Colombia

**Keywords:** Buried landmines, “Legbreaker” antipersonnel mines, Thermographic image analysis, Drone

## Abstract

This article presents a dataset of thermographic images of terrain with antipersonnel mines to identify the presence or absence of these artifacts using machine learning and artificial vision techniques. The dataset has 2700 thermographic images acquired at different heights, using a Zenmuse XT infrared camera (7-13 µm), embedded in the DJI Matrice 100 drone. The data acquisition experiment consists of capturing aerial infrared images of a terrain where elements with characteristics similar to antipersonnel mines type legbreaker were buried. The mines were planted in the ground between 0 cm and 10 cm deep and were spread over an area of 10 m x 10 m. The drone used a flight protocol that set the trajectory, the time of the flight, the acquisition height, and the image sampling frequency. This dataset was used in “Detection of “legbreaker” antipersonnel landmines by analysis of aerial thermographic images of the soil” [7].

**Specifications Table**

**Table:** 

Subject	Computer Science
Specific subject area	Computer Vision and Pattern Recognition
Type of data	JPG, TIFF, RJPG files
How the data were acquired	IR camera Zenmuse XT, on board UAV Matrice 100^1^ Weather station 2622330107 Rio Cañaveralejo, Melendez, Cali^2^ X: 1059854.91, Y: 868389.0697, Tipo: Automatic
Data format	Raw thermal images (720×480 pixels resolution), jpg format. Raw thermal images (336×256 pixels resolution), jpg format. Raw thermal images (336×256 pixels resolution), tif format. Temperature in°C degrees Irradiance in W/m^2^ Wind speed in m/s Daily precipitation in mm/d Mean Atmospheric Presure msnm Relative Humidity %
Description of data collection	The dataset was generated within the facilities of the Universidad of Valle, the images were taken by means of a drone and the acquisition schedules of the images were established according to previous studies in [1]. Images were taken from 1 to 10 m high for 9 areas where the deep of mine was varied (superficial, buried 1 cm, 5 cm, 10 cm and mine-free zone).
Data source location	Cali, Valle del Cauca, Colombia, South America
Data accessibility	Repository name: Test Images of Buried landmines Data identification number: Version 4 Direct URL to data: https://data.mendeley.com/datasets/732ngnf4r3
Related research article	Forero-Ramírez, J. C., García, B., Tenorio-Tamayo, H. A., Restrepo-Girón, A. D., Loaiza-Correa, H., Nope-Rodríguez, S. E., Barandica-López, A., & Buitrago-Molina, J. T. (2022). Detection of “legbreaker” antipersonnel landmines by analysis of aerial thermographic images of the soil. Infrared Physics and Technology, 125. https://doi.org/10.1016/J.INFRARED.2022.104307

## Value of the Data

• The image dataset can be used to detect the presence of elements similar to antipersonnel mines in a field inspected by aerial thermography.
• The dataset can be used to apply thermal contrast enhancement techniques and segmentation of regions of interest in the infrared spectrum.
• The data set can be used as training information for artificial intelligence models that detect buried antipersonnel mines or similar objects in thermographic images, by using images captured with height variations.
• The dataset allows the characterization of a terrain with the presence of antipersonnel buried landmines between 0 cm and 10 cm deep from the surface, by using images captured with height variations.

## 1 Objective

The data set consists of photographic images captured from a camera mounted on a drone. The images have not been subjected to calibration, denoising or other pretreatment. Nor has a calibration process been performed on the images, as this dataset is oriented to the use of mine detection techniques based on the thermal difference between the mine materials and the ground. Therefore, it is not necessary to know the correspondence between the thermal image reading and the actual temperature. The temperature gradient information is an alternative for the detection of anti-personnel mines buried at different depths. The database contains images captured at different heights of the drone flight, in order to determine the range of heights at which APLs could be detected.

## 2 Data Description

The dataset is generated for the detection of antipersonnel mines buried or located on the surface of terrain with a low percentage of vegetation and clay, by using the thermal behavior of the materials that interact in the experiment. The dataset is composed of 2700 thermographic images and the specifications of the equipment used to acquire the data are presented. On the other hand, presents the information on the dates and environmental conditions under which the samples were taken.

This dataset is oriented to projects that use variations in gray intensities in order to establish the differences between the mine and the soil. That is, it is not oriented to the precise temperature measurement of the object of interest. However, the caps with which the mines were built are black which would allow them to be used as a calibration pattern using the metadata contained in all the images published in the dataset (central temperature of the image, emissivity, reflected temperature, ambient temperature). Environmental values at the time of sampling are reported in [7].

**Table:** 

Main Folder	
Subfolder 1 (dd_mm_aaaa)	
Subfolder 2 (image format)	
Subfolder 3 (depth)	
File (Central Temperauture_CaptureHeight_Date)	

The set of images is organized in folders according to capture dates, image format, depth of the mines and height of the drone. The images contained in the dataset do not have any preprocessing or noise applied.

**Fig. 1:**
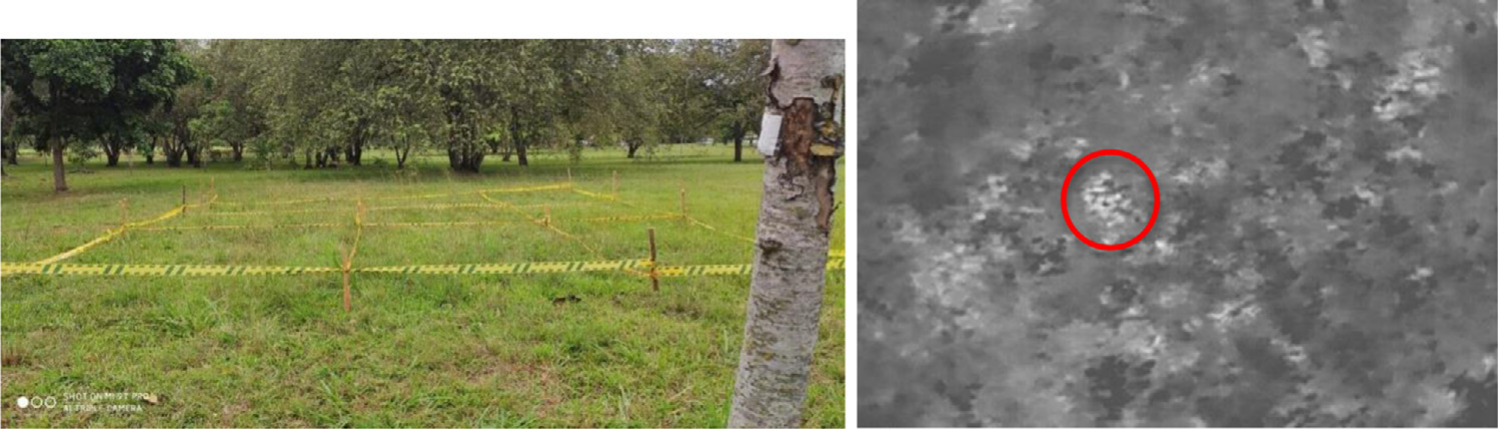
Picture of terrain to be inspected: visible spectrum (a); infrared spectrum zone 1 Mine 1cm deep Flight Altitude 3.9m and Temperature in center 34°C (b).

Fig. 1 is an image of the ground where the mines were buried. The only visible part of the mines is the top of the syringe plunger (used as a detonator), which is hidden by vegetation on the ground. The aim of this figure and the data set is to demonstrate that it is possible to locate mines in the infrared spectrum, whereas it is more difficult to do so in the visible spectrum. The infrared image was acquired by the Zenmuse XT thermal imaging camera on board the UAV Matrice 100.

The area was divided into nine rectangles zones: three zones (1, 2, 3) with a landmine at 1 cm depth, two zones (7, 8) with a landmine at 5 cm depth, one zone (9) with a landmine at 10 cm depth, two zones (4, 5) with a landmine arranged on surface, and one zone (6) free of landmines. The mines were buried in approximately half of each rectangular area in order to facilitate their segmentation in future work. The maximum depth to which a mine can be buried is 10 cm, and is given by the length of the plunger of the syringe used as a detonator.

## 3 Experimental Design, Materials and Methods

### 3.1 Materials

The materials used to build the database are detailed in four sections: Landmines, Soil of the Terrain, Image Acquisition System and Environmental Conditions Measuring Equipment:

### 3.2 Landmines

**Fig. 2:**
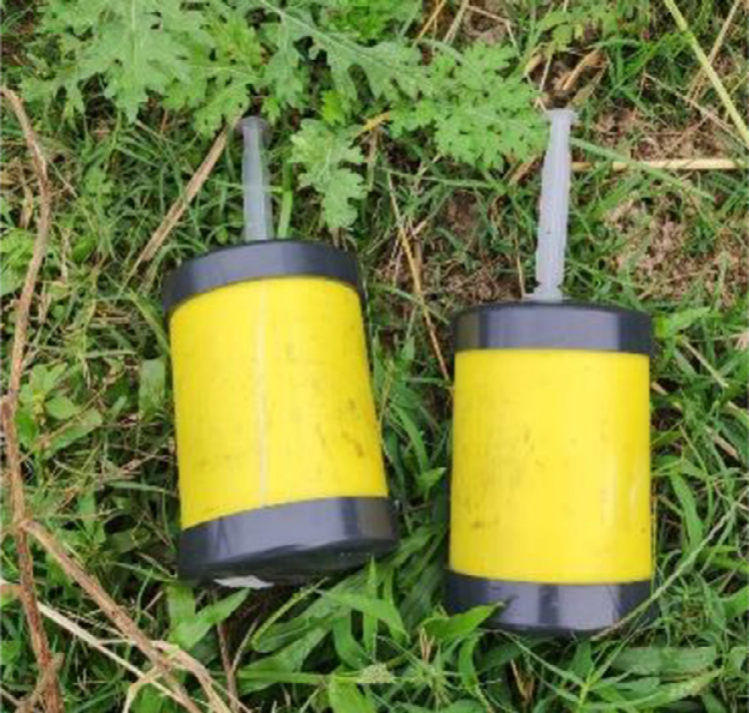
Structure of antipersonnel mines used in experiment.

The landmines (Fig. 2) were constructed using a PVC cylinder, a 5 ml syringe as detonator, 20 g of nails and 400 g of anthracite charcoal.

• PVC structure that simulates the antipersonnel mine according to the dimensions established by the Colombian national army [1] (Table 1).

**Table 1:** Structural specifications of the antipersonnel mine.

Diameter (m)	Height (m)	Plunger diameter (m)
0,087	0,102	0,014
• Anthracite coal has thermal characteristics similar to those of TNT [2,3], in terms of specific heat and thermal conductivity (Table 2). Anthracite coal was used for safety reasons during the experimentation.

**Table 2:** Difference between the thermal characteristics of TNT and anthracite coal.

	Density difference (g/cm^3^)	Specific heat difference (J/g K)	Thermal conductivity difference (W/m K)
TNT- Anthracite	0.19	0.11	0.03

### 3.3 Soil of the terrain

The soil used is non-saline, with a pH of 5.73 (moderately acidic), with an electrical conductivity in 1:2 solution of 0.13 (dS/m), with 35.6% sand, 35.4% clay and 29% silt. Soil characterization was carried out by the *Laboratorio de Aguas y Suelos Agrícolas* (LASA)^3^ of the *Universidad del Valle*.

### 3.4 Image acquisition system

The image acquisition system consists of a drone, a thermographic camera mounted on the drone and iPad.

• Zenmuse XT thermal camera, with a pixel resolution of 336×256, spectral range 7.5-13 µm, angular vibration range ± 0.03° and thermal sensitivity <50 mK.
• Matrice 100 drone is a quadcopter with 20 min of stationary flight time with full payload, 5 m/s maximum ascent rate, 4 m/s maximum descent rate and -10 to 40° operating temperature.
• Ipad with DJI Go^4^ application software to program the drone flight, monitor flight variables and define image capture times.

### 3.5 Environmental measuring equipment

The information on ambient temperature, solar radiation, relative humidity and wind speed was taken from the government weather station at Cañaveralejo^5^, which is the closest (approximately 3 km) to the terrain where the landmines were buried. The values of these variables on the days of thermography acquisition can be consulted in [7]. The environmental variables reported are those considered in other works of thermographic captures from a drone [8].

## 4 Method

**Fig. 3:**
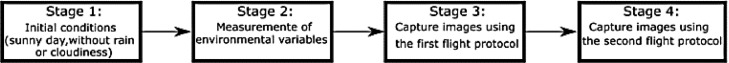
Images acquisition protocol.

An experimental protocol with 4 stages was designed to acquire the thermal images, as shown in Fig. 3.

STAGE 1:

As suggested in [1], the capture of minefield thermograms was performed on sunny, cloudless and rain-free days. Furthermore, following the recommendation of [2], the acquisition was performed between 17:00 and 18:00 hours. This period is suitable because there are thermal differences between the ground and the antipersonnel mine, which facilitates its detection.

STAGE 2:

The ambient temperature and wind speed are within the ranges suggested in [3], thus:

• Wind speed at 3-5 m/s, to guarantee the precision specification in temperature measurement calculated by the thermal camera sensor in this type of experiments.
• Ambient temperature at a range of 26-30°C, to favor the thermal diffusion of the antipersonnel mine and the ground surface.

STAGE 3:

In this phase, the drone is positioned approximately in the center of the zone to be inspected at a height of 10 m, to capture the first thermal image. Next, images are captured by decreasing the height of the drone by 1 m until it descends to 2 m. Thus, 9 images are obtained (in jpg and tiff formats) for each of the 9 zones into which the land to be surveyed was divided (a total of 81 images per zone).

STAGE 4:

In this phase, the drone descends to a height of 1 m above the inspection zone, where detection is facilitated by the higher resolution of the thermal gradient between the landmine and the soil [6]. At this altitude, 11 thermograms are acquired (one per second) to account for the change in the drone's position due to the wind, which added to the thermal noise of the camera, provides variability in the database. This procedure is repeated for each zone.

### 4.1 Experimental design

The experimental design considered variations in the depth at which landmines were buried (0, 1, 5 and 10 cm this is because it is the maximum length of the detonator or syringe), the distance between the camera (on board the drone) and the soil, and the image storage format. In addition, by performing the thermogram acquisitions on three different days, it was felt that the variability of environmental conditions more closely those that would be encountered in actual mine detection. Information on environmental conditions (irradiance, relative humidity, wind speed and ambient temperature) is presented in more detail in [7].

For the acquisition of each thermographic image, the camera was positioned perpendicular to each of the nine survey zones, as recommended by [4,5]. In addition, the images in the database contain information on the specifications of the camera, drone, gimbal and GPS parameters, as well as the configuration of their parameters during acquisitions, which may be of interest in the application of certain techniques. This information can be viewed using the Windows file explorer by right clicking on properties and then details. The distance between the soil and the camera was varied between 1 and 10 m (at 1 m intervals) to account for the effect of spatial resolution on mine detection.

## Ethics Statement

This dataset does not contain information involving humans, animal experiments or data collection from social media platforms.

## CRediT authorship contribution statement

**Hermes Alejandro Tenorio-Tamayo:** Conceptualization, Methodology, Software, Investigation. **Juan Camilo Forero-Ramírez:** Data curation, Writing – original draft, Investigation. **Bryan García:** Visualization, Investigation, Project administration, Resources. **Humberto Loaiza-Correa:** Supervision, Formal analysis, Writing – review & editing. **Andrés David Restrepo-Girón:** Supervision. **Sandra Esperanza Nope-Rodríguez:** Software, Validation, Writing – review & editing, Project administration. **Asfur Barandica-López:** Writing – review & editing. **José Tomás Buitrago-Molina:** Writing – review & editing.

## Data Availability

Test Images of Buried Landmines (Original data) (Mendeley Data).

## Footnotes

1 https://www.dji.com/matrice100.

2 https://ecopedia.cvc.gov.co/modulo-consulta.

3 https://eidenar.univalle.edu.co/laboratorios/laboratorios-de-aguas-y-suelos-agricolas.

5 https://ecopedia.cvc.gov.co/modulo-consulta

## Footnotes

4 https://www.dji.com/goapp.
